# Evaluating the variation in the projected benefit of community-wide mass treatment for schistosomiasis: Implications for future economic evaluations

**DOI:** 10.1186/s13071-017-2141-5

**Published:** 2017-04-28

**Authors:** Hugo C. Turner, James E. Truscott, Alison A. Bettis, Sam H. Farrell, Arminder K. Deol, Jane M. Whitton, Fiona M. Fleming, Roy M. Anderson

**Affiliations:** 1 London Centre for Neglected Tropical Disease Research, London, UK; 2 Department of Infectious Disease Epidemiology, School of Public Health, Faculty of Medicine, St Marys Campus, Imperial College London, Norfolk Place, London, W2 1PG UK; 3 Oxford University Clinical Research Unit, Wellcome Trust Major Overseas Programme, Ho Chi Minh City, Vietnam; 4 Centre for Tropical Medicine and Global Health, Nuffield Department of Medicine, University of Oxford, Oxford, UK; 5 Schistosomiasis Control Initiative, Department of Infectious Disease Epidemiology, School of Public Health, Faculty of Medicine (St. Mary’s Campus), Imperial College London, Norfolk Place, London, W2 1PG UK

**Keywords:** Schistosomiasis, Community-wide treatment, Mass drug administration, MDA, Modelling

## Abstract

**Background:**

The majority of schistosomiasis control programmes focus on targeting school-aged children. Expanding the use of community-wide mass treatment to reach more adults is under consideration. However, it should be noted that this would require a further increase in programmatic resources, international aid, and commitment for the provision of praziquantel. Consequently, it is important to understand (i) where a change of strategy would have the greatest benefit, and (ii) how generalisable the conclusions of field trials and analytical studies based on mathematical models investigating the impact of community-wide mass treatment, are to a broad range of settings.

**Methods:**

In this paper, we employ a previously described deterministic fully age-structured schistosomiasis transmission model and evaluate the benefit of community-wide mass treatment both in terms of controlling morbidity and eliminating transmission for *Schistosoma mansoni,* across a wide range of epidemiological settings and programmatic scenarios. This included variation in the baseline relative worm pre-control burden in adults, the overall level of transmission in defined settings, choice of effectiveness metric (basing morbidity calculations on prevalence or intensity), the level of school enrolment and treatment compliance.

**Results:**

Community-wide mass treatment was found to be more effective for controlling the transmission of schistosome parasites than using a school-based programme only targeting school-aged children. However, in the context of morbidity control, the potential benefit of switching to community-wide mass treatment was highly variable across the different scenarios analysed. In contrast, for areas where the goal is to eliminate transmission, the projected benefit of community-wide mass treatment was more consistent.

**Conclusion:**

Whether community-wide mass treatment is appropriate will depend on the local epidemiological setting (i.e. the relative pre-control burden in adults and transmission intensity), and whether the goal is morbidity control or eliminating transmission. This has important implications regarding the generalisability of cost-effectiveness analyses of schistosomiasis interventions. Our results indicate that areas with poor school-enrolment/coverage could benefit more from community-wide treatment of praziquantel and should potentially be prioritised for any change in strategy. This work highlights the importance of not over-generalising conclusions and policy in this area, but of basing decisions on high quality epidemiological data and quantitative analyses of the impact of interventions in a range of settings.

**Electronic supplementary material:**

The online version of this article (doi:10.1186/s13071-017-2141-5) contains supplementary material, which is available to authorized users.

## Background

Schistosomiasis, also known as snail fever or bilharziasis, is a neglected tropical disease (NTD) caused by parasitic flatworms belonging to the genus *Schistosoma*. There are two major forms of human schistosomiasis, intestinal and urogenital, which are caused by five main species of blood fluke. Schistosomiasis affects almost 240 million people worldwide, and more than 700 million people are at risk of infection [1]. At present, it is predominantly controlled by school or community-based mass drug administration (MDA) using praziquantel. The majority of schistosomiasis control programmes focus on targeting school-aged children (SAC), *via* school-based treatment programmes. In some areas, adults are also targeted, ranging from high-risk groups to the entire community [2]. The current World Health Organization (WHO) goals for schistosomiasis are outlined in Table 1.

**Table 1:** Overview of the WHO 2013 strategic plan for schistosomiasis [85]

Goals
1. To control morbidity due to schistosomiasis by 2020 (defined as 100% geographical coverage, 75% national coverage and < 5% prevalence of heavy-intensity infections across all sentinel sites).
*2.* To eliminate schistosomiasis as a public-health problem by 2025 (defined as a prevalence of heavy-intensity infections < 1% in all sentinel sites).
3. To interrupt transmission of schistosomiasis in the Region of the Americas, the Eastern Mediterranean Region, the European Region, the South-East Asia Region and the Western Pacific Region, and in selected countries of the African Region by 2025 (defined as reduction to zero incidence of infection).
Objectives
1. To scale-up control and elimination activities in all endemic countries
2. To ensure an adequate supply of praziquantel and resources to meet the demand

The global treatment coverage for schistosomiasis is the lowest of all the helminth diseases treated with preventive chemotherapy. Although it has improved recently, schistosomiasis remains classed as red by the annual score card developed by Uniting to Combat NTDs [3]. In 2014 the coverage of at risk SAC and adults was estimated to be 24 and 9%, respectively [4]. This is in spite of a recent increase in the availability of donated praziquantel (Merck KGaA has now increased its donation of praziquantel to up to 250 million tablets a year, equivalent to 100 million treatments [5]).

There is a growing body of evidence regarding the burden of infection and morbidity in adults, as well as the potential role of these age groups in sustaining transmission [4, 6–11]. This points to a greater need for inclusion of adults in schistosomiasis preventive chemotherapy treatment programmes in some endemic settings. While expanding the use of community-wide mass treatment to reach more adults is under consideration [12, 13] this would require a further increase in programmatic resources and international commitment. The main bottleneck to schistosomiasis control efforts at present is not the availability of donated praziquantel, but resources and funds for its delivery (especially in remote and hard to reach settings) [14].

Although several field studies have been conducted to investigate the benefit of community-wide mass treatment for schistosomiasis control, findings have been inconsistent [15–18] and some studies find no significant difference when comparing its impact to school-based treatment. Furthermore, Butterworth and colleagues [19] performed studies in Kenya, comparing the long-term impact of different methods of administration of chemotherapy (selective treatment to all infected individuals, selective treatment to individuals with heavy infections, and selective treatment of infected school children). The arm performing selective treatment of all infected individuals showed the greatest relative reductions in infection prevalence and intensity (however, some earlier interventions had been carried out in this area and the pre-treatment intensities were lower than the other arms). The arm providing treatment only to infected schoolchildren also had a marked and prolonged effect (which was comparable to - if not better than - selective treatment of individuals with heavy infections). It was concluded that, in areas of low morbidity, chemotherapy of SAC alone is a satisfactory way of producing a long-term reduction in both infection intensity and morbidity [19].

**Fig. 1:**
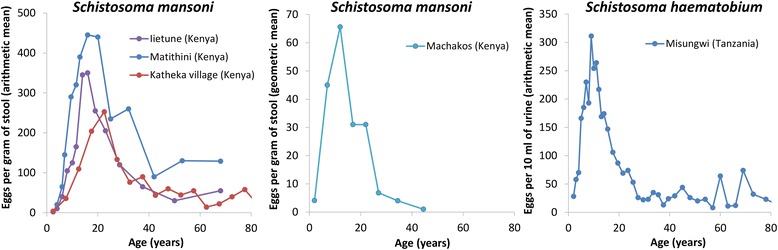
The observed cross-sectional host-age and mean intensity profiles for *Schistosoma mansoni and Schistosoma haematobium* infection. The data are from the following sources: Iietune (Kenya) [64], Matithini (Kenya) [64], Katheka village (Kenya) [86], Machakos (Kenya) [87], Misungwi (Tanzania) [88]

It is important that we understand the variability of the impact of using community-wide mass treatment across different settings, and in what circumstances it has the greatest benefit (particularly as the relative pre-control burden in adults, has been observed to vary across different settings (Fig. 1) [20]). In this study, we use a mathematical model to evaluate the projected benefit of using annual community-wide mass treatment in terms of both controlling morbidity and eliminating transmission for *Schistosoma mansoni* (the most prevalent of the schistosome species infecting humans). We investigate how sensitive this projected benefit is to different epidemiological and programmatic assumptions. We also explore how the benefit of switching to community-wide mass treatment is influenced by the chosen effectiveness metric, i.e. what method is used to approximate MDA’s impact on morbidity. This is important as there is uncertainty regarding how schistosomiasis models should approximate the impact of treatment on morbidity. The principle goal of this research is to gain an understanding of how generalisable the conclusions of studies investigating the impact and benefit of community-wide mass treatment are across different settings.

## Methods

### Mathematical model

**Fig. 2:**
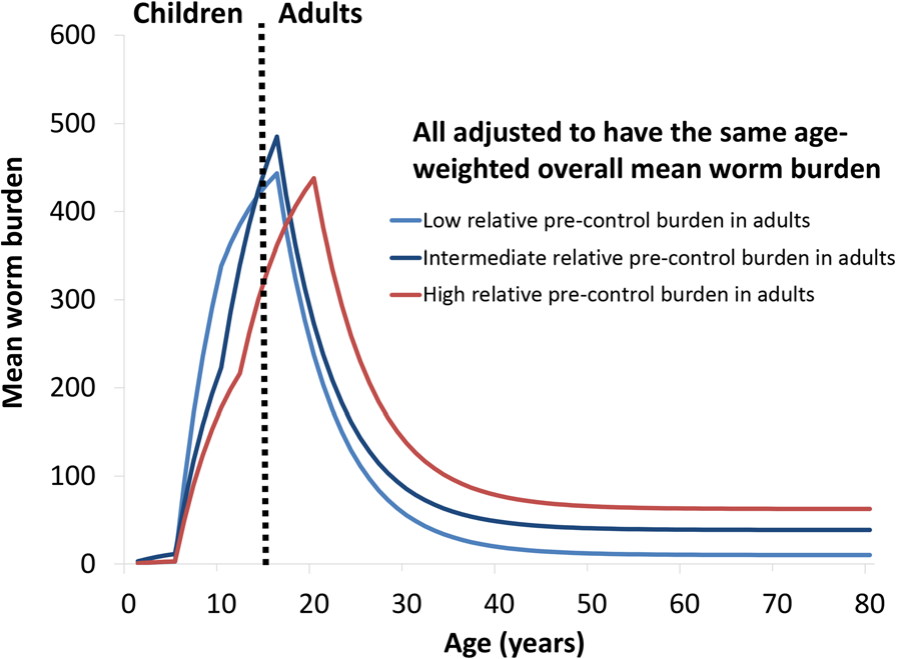
Model scenarios regarding the age-intensity profiles of infection and the relative pre-control burden in adults. To ensure the results are comparable, the R_0_’s were adjusted for these different profile scenarios such that they had the same pre-control mean worm burden (i.e. we ensured that we are not comparing the impact of both a different age-profile and a different initial overall burden). Model parameters are shown in Additional file 1: Tables S1 and S2

In this paper, we employ a previously described deterministic fully age-structured schistosomiasis transmission and MDA treatment model [4, 21], which assumes the parasite is dioecious and monogamous, has density-dependent egg production [22] and a degree of parasite aggregation defined by the negative binomial probability distribution with a fixed *k* value. The model describes changes in worm burden in order to capture the non-linear and density dependent processes which influence the effect treatment has on the rate of transmission, such as the worm’s mating behaviour and the effect of infection intensity on the female worm’s fecundity. The predictions of MDA impact generated by this model are very similar to a more complex individual-based stochastic model [6]. In the model, we assume that the age-intensity profiles (Fig. 2) are generated by age-dependent exposure to the infectious stages in the environment and not acquired immunity [23]. Although the model has full age structure, the outputs are grouped into programmatically meaningful categories such as SAC (5–14 year- olds) and adults (≥15 year-olds)*.* The response of the model under treatment has been validated against data (see [4, 6] for further details). The model has been adapted to allow for systematic non-compliance (individuals never taking treatment) [24]. Further detail regarding the model and its parameters is provided in Additional file 1.

### Model scenarios and sensitivity analysis

We investigated the impact of an annual school-based treatment programme targeting only SAC, and compared this with an annual community-wide mass treatment programme targeting both SAC and adults. Based on a recent systematic review, we assumed the drug efficacy of a 40 mg per kg dose of praziquantel was 86.3% for *S. mansoni* [25]. In line with WHO guidelines, we assumed Pre-SAC were not eligible for treatment [26].

We assumed a 75% treatment coverage of the targeted age groups and a systematic non-compliance rate of 5%, i.e. 5% of the targeted population never take treatment, and the rest of the eligible population take it randomly each round. It should be acknowledged that there are very little data regarding systematic non-compliance rates [27] and we, therefore, varied these assumptions in the sensitivity analysis.

We also considered a scenario where the systematic non-compliance rate was 20% for the school-based programme, but 5% when using community-wide mass treatment, simulating a setting where many of the non-enrolled SAC are consistently missed by the school-based programme, and only reached through a community-based programme.

Based on the available age-intensity profiles (Fig. 1) [6], we defined three scenarios with different levels of relative pre-control infection burdens in adults (low, medium and high, see Fig. 2 and Additional file 1: Tables S1 and S2). These were chosen to represent the observed variation in the relative burden in adults and were based on the fits presented in [6]. It should be noted that these are informed by the limited age-stratified epidemiological data available on infection intensity, and consequently, there may be settings that fall outside of this range. The model was used to simulate two transmission settings: a higher transmission setting with an overall age-weighted mean worm burden of 155, based on model fits to the data [4], and a lower transmission setting with a mean worm burden of 60. The age-weighted mean egg count per gram of stool (epg) varied between 158 and 166 for the higher transmission setting and 76–78 for the lower transmission setting. The age-weighting accounts for the local demography. To ensure the results for the different scenarios are comparable, the transmission intensity, as represented by the basic reproduction number (R_0_), was adjusted such that the different scenarios were based on pre-control mean worm burden, i.e. we ensured that we are not comparing the impact of both a different age-infection profile and a different pre-control burden when comparing the different scenarios. The R_0_ values ranged between 1.30 and 1.32 for the lower transmission setting and 1.63–1.68 for the higher transmission setting.

The sensitivities of the model projections to the assumed life expectancy of the adult worms (4 years instead of 5.71), and the treatment coverage and compliance levels were explored.

### Effectiveness metrics

**Fig. 3:**
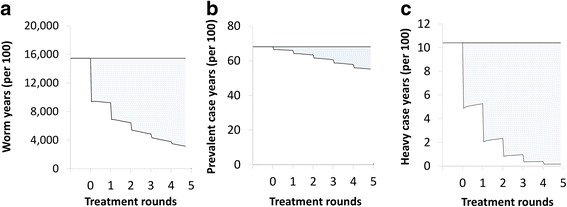
Schematic representation of the effectiveness metrics. **a** Reduction in the overall worm burden (worm years averted). **b** Prevalent infection case years averted. **c** Heavy case years averted. Heavy burden was defined as an epg ≥ 400. The total effectiveness was the total shaded area across the full-time horizon. The metrics are described in more detail in [76]

The model was used to investigate three different effectiveness metrics (modified from [28]) across the chosen time horizon (Fig. 3): (i) the total reduction in the overall worm burden experienced by the population, i.e. number of years lived with a worm (worm years) averted; (ii) the total number of prevalent infection case years averted, i.e. the number of years lived with a prevalent infection prevented; and (iii) the total number of heavy infection case years averted, i.e. the number of years lived with a heavy infection prevented. Heavy infection was defined as having an epg ≥ 400, an established WHO threshold [2].

We also estimated the number of rounds of preventive chemotherapy required to eliminate transmission - defined as crossing the breakpoint in transmission where infection levels settle to the equilibrium of extinction [29].

## Results

### Impact of school-based treatment

**Fig. 4:**
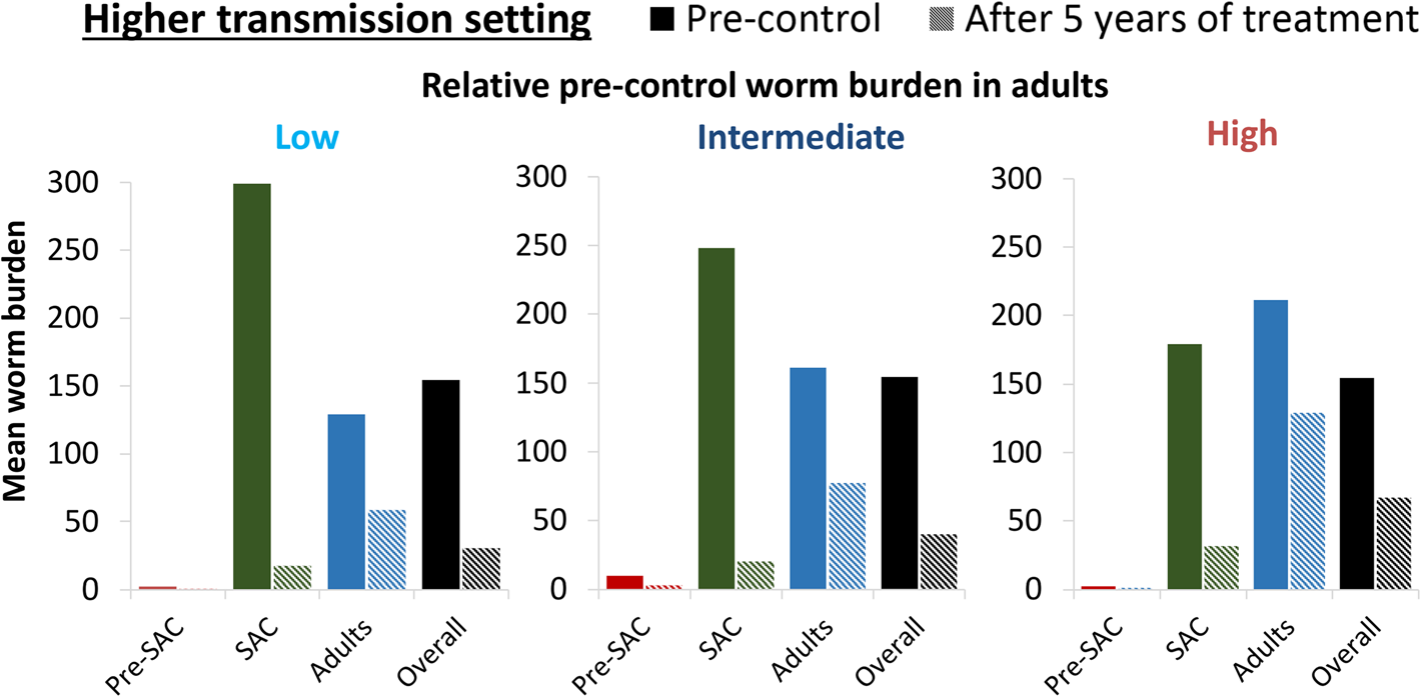
The impact of annual school-based treatment on the mean worm burden in different age groups. The solid bars represent the pre-control burden and the hashed bars, the burden after 5 years of treatment. The scenarios for the relative pre-control burden in adults are shown in Fig. 2 (note they have the same age-weighted overall mean worm burden). The results assume a treatment coverage of 75% and 5% systematic non-compliance. The results pertaining to the lower transmission setting are shown in Additional file 1: Figure S1. *Abbreviation*: Pre-SAC: 2–4 year-olds, SAC: 5–14 year-olds and adults: ≥ 15 year-olds

**Fig. 5:**
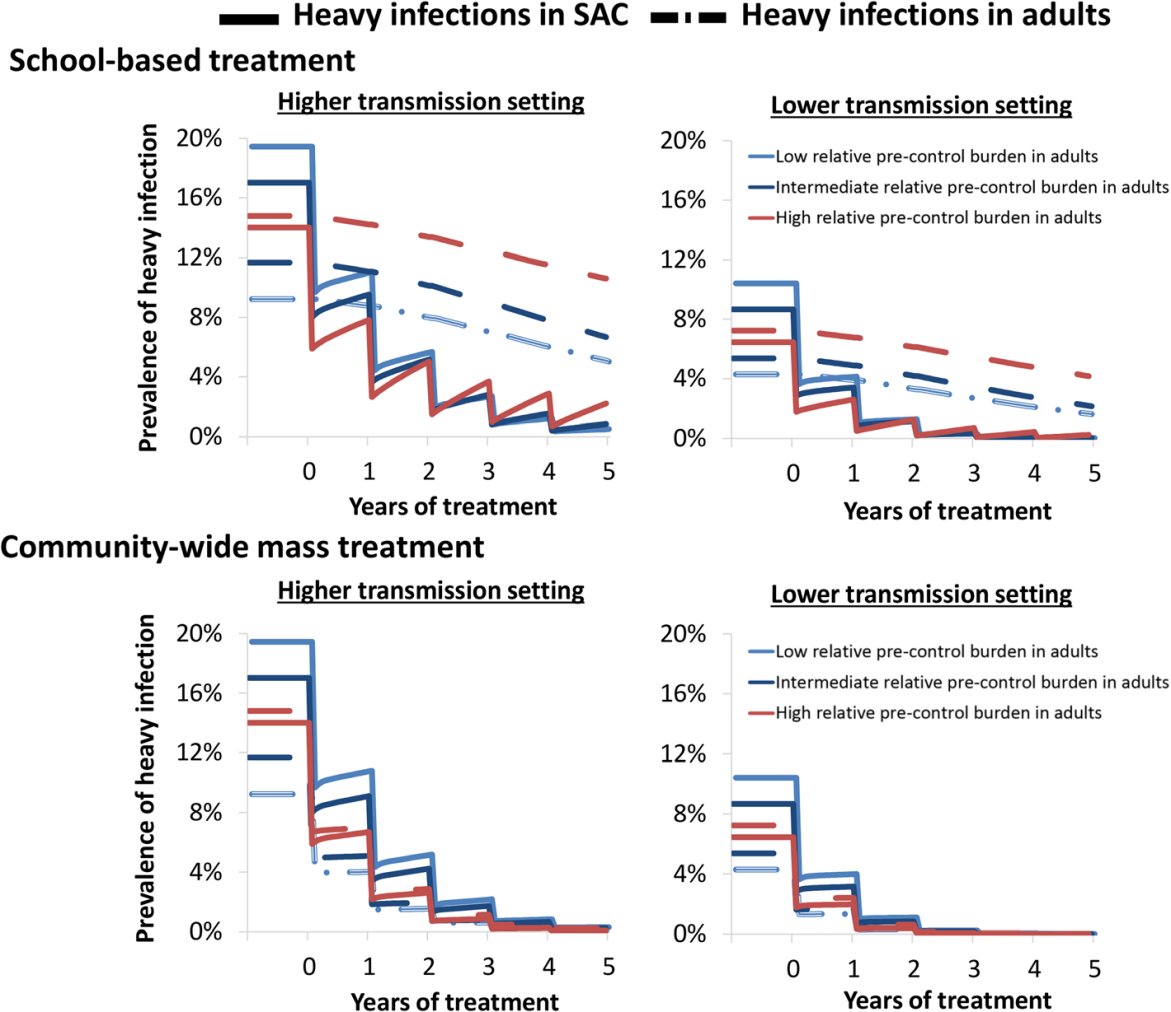
The impact of annual school-based treatment and community-wide mass treatment on the prevalence of heavy infections in SAC and adults. The solid lines represent the prevalence of heavy infection in SAC and the dashed the prevalence of heavy infection in adults. The different scenarios for the relative pre-control burden in adults are indicated with different coloured lines (see Fig. 2). Heavy infection was defined as having a burden above the WHO threshold (≥400 epg) [2]. The results assume a treatment coverage of 75% and 5% systematic non-compliance. *Abbreviation*: SAC: 5–14 year-olds and adults: ≥ 15 year-olds

Annual school-based treatment was projected to notably reduce both the worm burden and prevalence of heavy infections in SAC. It also has an indirect effect on the untreated adults due to reductions in transmission (Figs. 4 and 5). However, the overall impact of school-based treatment was found to be dependent on the relative pre-control burden in adults (the shape of the age intensity of infection profile) and in many settings a significant burden would remain even after five years of treatment (Figs. 4 and 5). The higher the level of transmission, the greater the significance of this untreated burden in adults (Figs. 4, 5 and Additional file 1: Figure S1).

### Benefit of community-wide mass treatment stratified by effectiveness metric

A community-wide mass treatment strategy was found to increase the effectiveness of preventive chemotherapy against schistosomiasis, though its benefit relative to school-based treatment was found to vary across the different epidemiological scenarios and the employed effectiveness metrics used to quantify the intervention’s impact on morbidity (Table 2). The main prediction was that scenarios with a higher pre-control burden in adults have a greater relative benefit of using community-wide mass treatment (Table 2). The estimated relative increase in effectiveness was highest when using prevalent case years averted as the effectiveness metric, and was considerably lower in many settings when using the metrics based on infection intensity (either worm years or heavy infection case years averted). For example, when assuming a relatively low pre-control burden in adults, the increase in the number of worm-years averted when using community-wide mass treatment was only 15–17%, in contrast to an increase of 70–83% for the number prevalent case years averted.

**Table 2:** Projected incremental increase in effectiveness when using annual community-wide *versus* school-based treatment

Effectiveness metric	Relative pre-control worm burden in adults	Incremental increase in effectiveness (relative increase in effectiveness)	
		Higher transmission setting	Lower transmission setting
Average number of worm-years averted per person	Low	306 (17%)	107 (15%)
	Medium	481 (30%)	155 (23%)
	High	948 (80%)	314 (59%)
Prevalent case years averted (per 100 individuals)	Low	146 (83%)	176 (70%)
	Medium	168 (124%)	223 (99%)
	High	237 (303%)	305 (258%)
Heavy case years averted (per 100 individuals)	Low	27.1 (23%)	8.1 (12%)
	Medium	44.5 (43%)	11.4 (18%)
	High	85.5 (118%)	26.7 (52%)

The scenarios for the relative pre-control burden in adults are shown in Fig. 2 (note they have the same age-weighted overall mean worm burden). The results assume a treatment coverage of 75% and 5% systematic non-compliance. The analysis was conducted with a five-year implementation period and a 15-year time horizon (i.e. looking at the effect of five years of treatment for 15 years)

The incremental increase in effectiveness in terms of worm years and heavy infection case years averted was larger in high transmission settings (Table 2). However, the incremental increase in the number of prevalent case years averted was higher in lower transmission settings (Table 2).

In high transmission settings, we found that using community-wide treatment was not only more effective for reducing the overall burden of heavy infections but also more effective for controlling the prevalence of heavy infections specifically in children (Fig. 5).

#### Sensitivity analysis

The key results were found to be robust with respect to the sensitivity analyses performed - though the precise estimated benefit of community-wide mass treatment showed some variation (Additional file 1: Tables S3-S6). Of particular note was, the projected benefit of switching to community-wide mass treatment decreased when assuming a lower coverage of adults (Additional file 1: Table S3). In contrast, when assuming a scenario where community-wide treatment would decrease the level of systematic non-compliance in SAC, its benefit increased (Additional file 1: Table S6).

### Impact on transmission and projected rounds to elimination

**Fig. 6:**
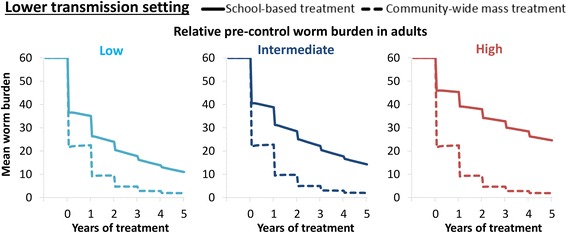
Comparison of the impact of annual school-based treatment and community-wide mass treatment on the overall mean worm burden. The scenarios for the relative pre-control burden in adults are shown in Fig. 2 (note they have the same age-weighted overall mean worm burden). The results assume a treatment coverage of 75% and 5% systematic non-compliance

**Fig. 7:**
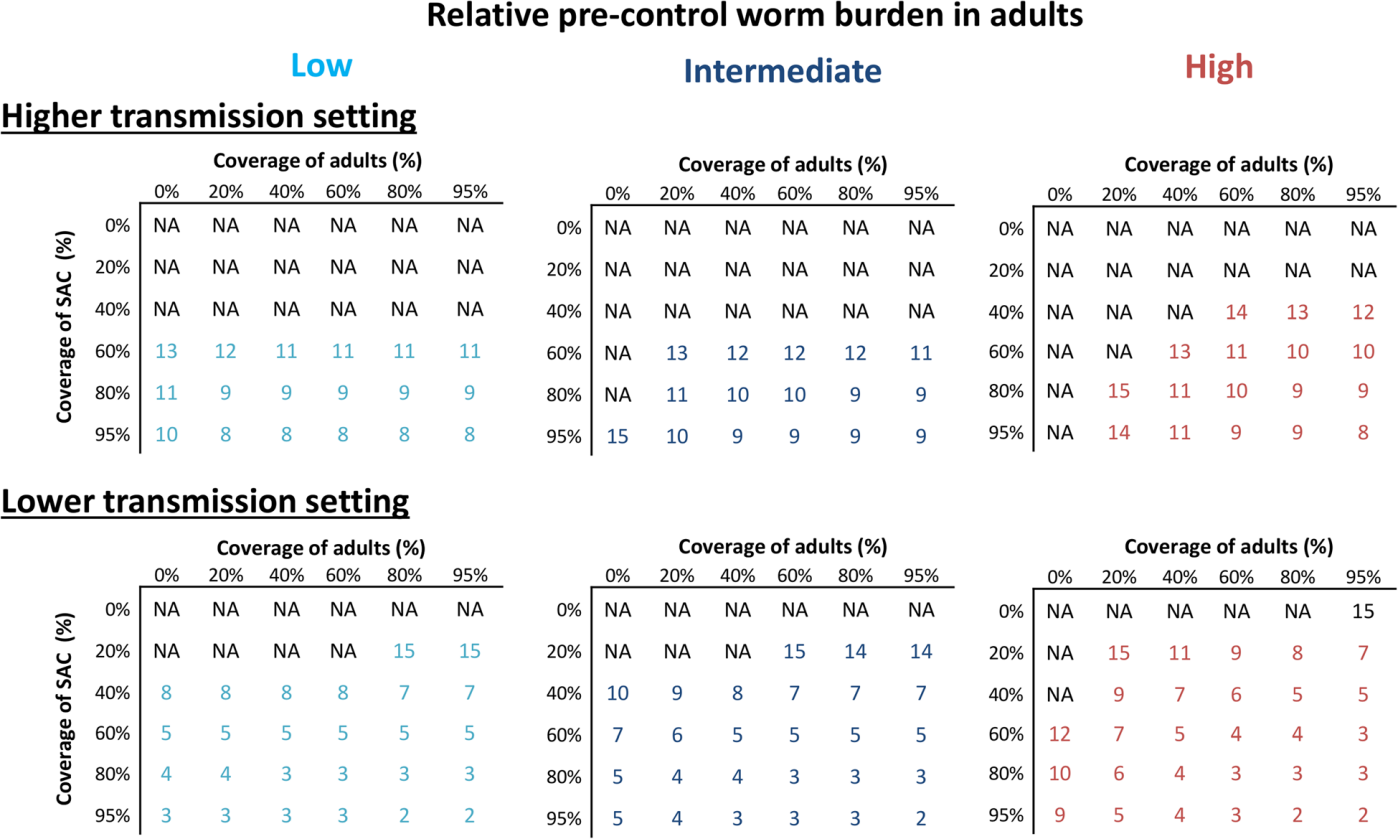
The projected number of years of annual treatment required to achieve elimination of *Schistosoma mansoni*. The scenarios for the relative pre-control burden in adults are shown in Fig. 2 (note they have the same age-weighted overall mean worm burden). The results assume 5% systematic non-compliance (and therefore the coverage cannot be higher than 95%). *Abbreviation*: NA, not achievable within 15 years of annual treatment

The impact of school-based treatment on the overall level of transmission varied in the different scenarios. However, even when assuming a relatively low pre-control burden in adults and a high coverage of SAC, the reservoir in the untreated adults and SAC still had important implications for the level of ongoing transmission (Fig. 6). In other words, even though these lower intensity infections may not always be sufficient to justify community-wide mass treatment when focussing on morbidity control, they can become significant when trying to break transmission (Fig. 6). These results are mirrored when looking at the number of rounds required to break transmission (Fig. 7) and suggest that when the goal is eliminating transmission, community-wide mass treatment is the best strategy in most settings. However, the results indicate that in high transmission settings it may not be feasible to break transmission with annual preventive chemotherapy alone (Fig. 7 and Additional file 1: Figure S2) [4, 6].

#### Sensitivity analysis

When assuming a higher rate of systematic non-compliance (such as 20%), achieving elimination was projected to be less feasible, requiring more treatment rounds and higher coverage rates (Additional file 1: Figure S2). If a lower mean life expectancy of the adult worms was assumed during the model fitting (4 years instead of 5.71), the projected number of rounds to elimination was generally slightly higher, though the overall findings remained consistent (Additional file 1: Figure S3).

**Fig. 8:**
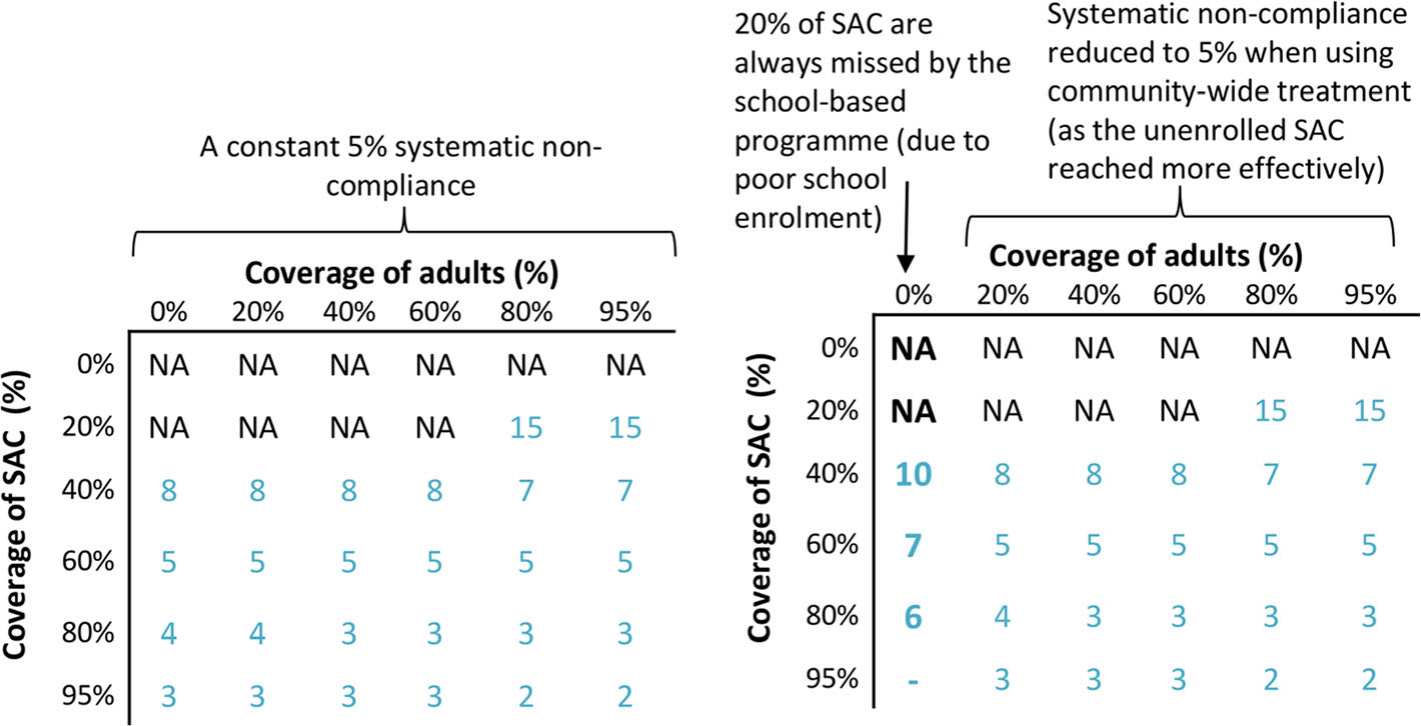
Potential impact of annual community-wide mass treatment decreasing the rate of systemic non-compliance in school-aged children. The results pertain to the scenario with a relatively low pre-control worm burden in adults (Fig. 2). When assuming a systematic non-compliance rate of 20%, it is not possible to get a 95% coverage of SAC (indicated by a dash in the figure). *Abbreviation*: NA, not achievable within 15 years of annual treatment

The results presented in Fig. 7 and Additional file 1: Figures S2-S3 assumed that the rate of systematic non-compliance was the same, for both school-based and community-based treatment programmes. However, it is possible that expanding treatment from schools into the community to reach adults could also increase the number of SAC regularly receiving treatment (as non-enrolled SAC could also be reached more effectively). In such circumstances, the benefit of expanding treatment into the community for eliminating transmission would be greater (Fig. 8).

## Discussion

### The benefit of community-wide mass treatment

Community-wide mass treatment was found to be more effective for controlling the transmission of schistosome parasites, than using a school-based programme only targeting SAC. However, in the context of morbidity control, the potential benefit of switching to community-wide mass treatment was variable across the different scenarios explored. The benefit was found to be highly dependent on the transmission intensity, the level of school enrolment/compliance to treatment, and the relative pre-control worm burden in adults. This has important implications regarding the generalisability of any cost-effectiveness analysis of schistosomiasis interventions. The results indicate that in some settings the indirect benefits of a school-based programme on the untreated adults may mean that community-wide mass treatment is unlikely to be more cost-effective in terms of preventing morbidity. This implies that, in settings where the goal is morbidity control, the best strategy may be to scale-up the geographical coverage of the school-based programmes (which is currently low in many settings [30]) and prioritise community-wide mass treatment in settings where the burden in adults and transmission intensity are known to be high, or where school enrolment is poor.

It is important to note that though regular annual community-wide mass treatment may not always be advisable for morbidity control, this does not mean that high-risk adults should not be targeted when possible, particularly pregnant women [31, 32] and those in high-risk occupations, such as fishermen. Where resources are too limited to permit expansion to community-wide mass treatment, a potential solution could be to further encourage treatment of the parents of SAC within the school-based programmes or through other existing platforms such as Child Health Days.

Longer term, there is a shifting emphasis towards transmission elimination by WHO. With this aim, our analyses clearly show a benefit of community-wide mass treatment in most situations and it indicates that it would be required in order to succeed within a feasible time frame in most settings - with the exception of low transmission settings where there is little infection in adults. However, the projections also indicate that in high transmission settings it may not be possible to break transmission using annual rounds of preventive chemotherapy alone, and using other strategies such as increasing the treatment frequency, health education, WASH, and snail control, should also be considered [4, 6, 12]. When evaluating the cost-effectiveness of incorporating these alternative strategies, it will be important to consider the benefit of preventing hotspots reseeding the infection to other areas and communities.

### School-enrolment

School enrolment rates vary considerably both within and between sub-Saharan African countries. For example, UNICEF reports a number of settings where the net attendance rates are lower than 70% (with some as low as 21%), as well as a large disparity between urban and rural areas [33]. It is also important to consider that children may enrol in primary school but, due to unpaid school fees or seasonal work have to drop out [33]. For example, in Malawi 93% of children will enrol in primary school; however, only 48% will complete it [33]. Consequently, in the most marginalised and rural communities within a country very few children may be completing primary school.

Using a community-wide mass treatment strategy would likely improve the coverage and compliance of non-enrolled SAC - who can be missed when only using a school-based strategy. Analyses indicate that this can have notable implications for the benefit of switching to community-wide mass treatment for both morbidity control and eliminating transmission (Fig. 8 and Additional file 1: Table S6). This further highlights that areas with poor school-enrolment/coverage (or with high school dropout rates) should be prioritised for any shift to community-wide treatment.

### Variation in the observed and predicted impact of community-wide mass treatment

A fundamental reason why studies [15–18] find contrasting results regarding the benefit of community-wide mass treatment is the variation in the relative worm burden harboured by adults across different geographical settings; the higher the pre-control burden in adults, the larger the benefit of switching to community-wide treatment (Fig. 1). A second and often overlooked reason is the way the trial or model is implemented. For example, the age grouping used for the different treatment categories, how the data are categorised, and the time horizon for the analysis, can all influence the estimated strength of the indirect benefit of school-based treatment on the untreated adults. Consequently, these influence the benefit of switching to community-wide mass treatment.

**Fig. 9:**
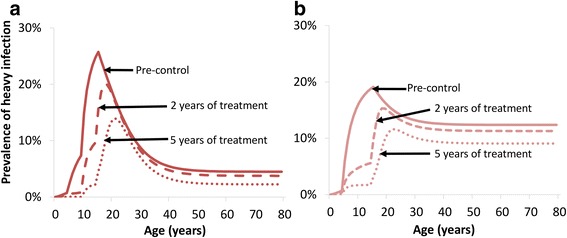
Modelling the indirect benefit of annual school-based treatment on the prevalence of heavy infection. **a** The model was fitted to fully age-structured data (and therefore accounts for the true shape of the age-intensity profile) [4]. **b** The model was only fitted to reproduce the estimated mean pre-control worm burdens in SAC and adults from the same dataset. The data are from the Iietune village (Kenya) [64] (Fig. 1). The results assume a treatment coverage of 75% and no systematic non-compliance

When evaluating different interventions against schistosomiasis, it is vital to account for the shape of the age-intensity profile prior to control. This is illustrated in Fig. 9, which compares the model’s projected impact of school-based treatment when (a) the model is fitted to fully age-structured data, which therefore accounts for the true shape of the age-intensity profile, and (b) the model is only fitted to the mean burdens of the SAC and adult age groups, i.e. using the summary statistics from the same dataset for these two age classes. This shows that if the shape of the infection profile is not accounted for, the burden of infection in different age groups and the impact of different interventions can be incorrectly quantified (Fig. 9). Specifically, not accounting for the infection profile’s shape can lead the model to underestimate the impact of school-based treatment (Fig. 9). In the example shown in Fig. 9, the more simple fitting method would result in the model overestimating the long-term incremental effectiveness of community-wide mass treatment between 29 and 42% for the different metrics investigated (Additional file 1: Table S7). This highlights the importance of using fully age-structured models in analyses investigating the impact of targeting different age groups, particularly for cost-effectiveness analyses. The difference between the two fitting methods will vary across different settings and will be dependent on the shape of the infection profile.

We would also like to highlight that it is important to understand in which individuals the burden of heavy-intensity infections remains after school-based treatment. For example, Fig. 9 shows that for the modelled setting, the majority of heavy infections remaining are in 20–30 year-olds. If a treatment intervention could be targeted at this remaining high-risk group, expanding to the whole community could be unnecessary for morbidity control in some settings, allowing resources to be redirected to improving coverage and compliance.

### Effectiveness metrics and morbidity control

The benefits of switching to community-wide mass treatment for schistosomiasis morbidity control were found to be very dependent on the choice of the effectiveness metric. i.e. what method has been used to approximate treatments impact on morbidity.

**Fig. 10:**
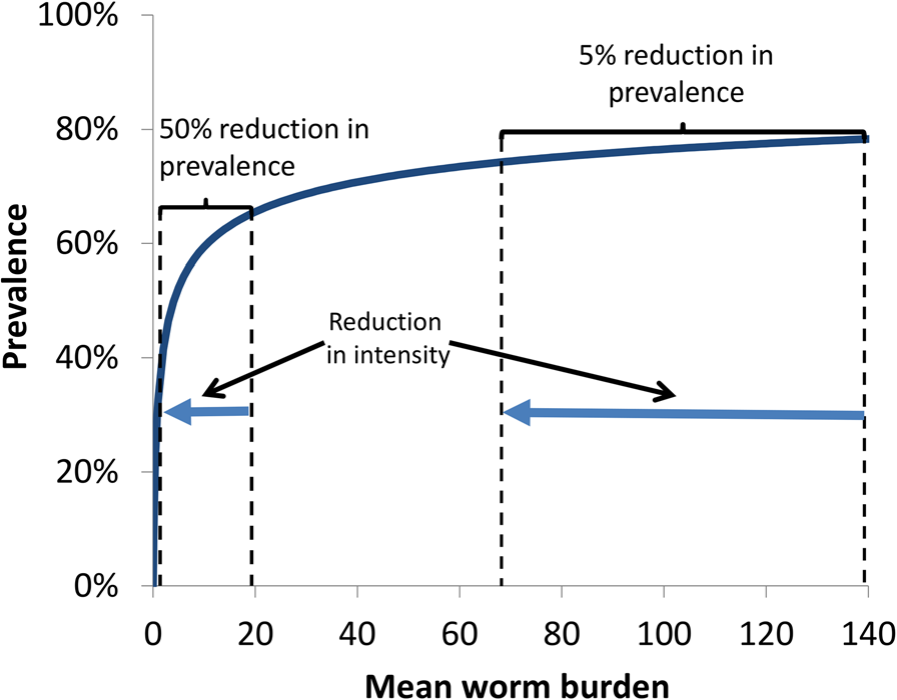
The relationship between infection intensity and prevalence. The relationship is further described in [6, 29]

The Disability-Adjusted Life Year (DALY) burden of schistosomiasis is often calculated by simply applying a disability weight, representing the disability of an ‘average’ prevalent case of schistosomiasis, to the prevalence of infection. However, it is important to note that the aim of disease burden studies (such as the global burden of disease (GBD) [34]) is to approximate the disease burden at a given point in time. We believe that for schistosomiasis it is misleading to apply this same framework to estimate the morbidity averted over time due to an intervention, i.e. calculating the number of DALYs averted by applying a disability weight to the number of detectable prevalent-case years averted. This is because the morbidity associated with schistosomiasis is complex and often not due merely to the presence or absence of infection [35–50]. Even the early stages of schistosomiasis-related morbidity (such as diarrhoea, anaemia, and calorie undernutrition), have been found to have a relationship (at least in part) to the individual’s intensity of infection [46–51]. This is important since when morbidity is related to the intensity of infection, estimating the impact of treatment on morbidity based solely on reductions in infection prevalence may result in a misleading quantification, particularly regarding the impact of treating different age groups. The key reasons for this are as follows:

(i) Considering only reductions in prevalence assumes that all infections are equally pathogenic and that reducing the intensity of someone’s infection but not curing it, has no health benefit. This is particularly significant for this research question as infection intensity tends to decrease in older age groups (Fig. 1). Furthermore, if infections are more pathogenic in children, estimating reductions in morbidity based on reductions in prevalence alone could overestimate the benefit and cost-effectiveness of switching to community-wide treatment. It should be noted that a recent systematic review and meta-analysis concluded that reductions in egg output are significantly correlated with decreased schistosomiasis-related morbidity [52].
(ii) Due to the nonlinear relationship between infection intensity and prevalence, treatment at high-intensity levels can result in a large reduction in average infection intensity but have only a small impact on prevalence (Fig. 10 [29]). Conversely, at lower intensity levels a small impact on infection intensity will lead to a dramatic reduction in prevalence (Fig. 10).

The nonlinear relationship between infection intensity and prevalence (Fig. 10) is why the number of prevalent case years averted was projected to be higher in the lower transmission setting by our model. This would imply that when modelling reductions in morbidity based on reductions in prevalence, the results could find that it is more cost-effective to treat in the lower transmission settings. The other investigated metrics found the opposite relationship and a greater impact in higher transmission settings.

When assessing studies evaluating the burden of schistosomiasis it is also important to consider that late stage morbidity may be permanent (or at least not cured by praziquantel treatment). This means that it is likely that at least some of the morbidity in adults will not be resolved by treatment - as it may be a consequence of the infection burden they had throughout childhood. Previous modelling studies have successfully captured trends in morbidity data using functions which relate morbidity to the accumulated past experience of infection [53–57]. The 2010 GBD study now includes separate calculations for the more advanced forms of schistosomiasis-related morbidity, such as hepatic inflammation, hematemesis, and ascites [58]. However, it is important to note that it does not include advanced urogenital diseases, infertility, or the late effects of growth stunting and cognitive impairment [58].

#### Burden of light infections

An important area of uncertainty regarding the benefit of community-wide mass treatment is the potential morbidity associated with active light infections; if their morbidity is high it would increase the benefit of community-wide mass treatment. These will be more frequently identified in field epidemiological studies with the new diagnostic tests (such as the CCA assay [59]) which have a greater sensitivity than egg counts in stool or urine. It is becoming increasingly recognised that the potentially subtle morbidity that results from low-intensity infections may be greater than previously thought and that even low-intensity infections may limit the productivity and reduce the wellbeing of infected adults [46, 52, 58, 60–62]. The potential burden of these light infections has important implications for the need to shift towards the elimination of transmission [62]. However, although light intensity infections may lead to morbidity [46, 52, 58, 60, 61], it is likely that they are more pathogenic in children than adults. Consequently, we believe that to account for the benefit of preventing these active light infections accurately, it is necessary to have a framework which:

(i) Accounts for the differences in the likelihood of morbidity in children versus adults at different levels of infection intensity, i.e. how pathogenic different levels of infection are in different age groups.
(ii) Accounts for which forms of morbidity are permanent (and not cured by treatment).

Without this, any conclusions regarding the benefit of expanding treatment on morbidity would be very dependent on assumptions that are based on limited empirical evidence. Overestimating the relative burden of light infections could overestimate the benefit and cost-effectiveness of switching to community-wide treatment.

It should be stressed that debate continues about whether there is a recognised infection intensity or infection duration threshold below which the risk for disease from *Schistosoma* infection becomes negligible [60, 61].

The potential morbidity of light infections is also very important regarding the benefit of a paediatric formulation of praziquantel [63]. Previous modelling has suggested that this would be of limited use in bringing about transmission elimination [4]. However, depending on the pathogenicity of these light infections in early childhood, a paediatric formulation of praziquantel could still be highly beneficial for morbidity control.

#### The most appropriate effectiveness metrics

The current gaps in knowledge in this area mean that it is difficult to accurately capture the impact of treatment on the morbidity related to schistosomiasis within transmission models. We believe that caution should be employed when interpreting modelling results in this area - which is the reason we did not attempt to estimate DALY averted within this analysis. In our opinion, worm-years (which acts as a metric of the cumulative experience of the population, Fig. 3) and the prevalence of heavy infections are currently the most informative metrics for evaluating the impact of different interventions on schistosomiasis-related morbidity within models. However, as the evidence in this area evolves and more data becomes available, the choice of effectiveness metric should be reassessed/modified, with the concomitant development of frameworks that can accurately estimate the number of DALYs averted.

### Limitations in the model projections

The model predictions reported in this paper were parameterised for *S. mansoni*. However, given the similar estimates of the life expectancies for other *Schistosoma* species [22, 64] the overall conclusions should be applicable to other species where humans are the dominant host in maintaining transmission.

It should be stressed that the field of schistosome epidemiology suffers greatly from limited information on key parameters such as detailed age-intensity profiles, and the relationship between egg output and worm burden [6]. An important area of uncertainty in the model projections is whether or not acquired immunity against the different schistosome species exists and to what extent it shapes the observed age-intensity profiles [6]. If a species was to generate a strong acquired immunity response, repeated rounds of preventive chemotherapy would reduce the level of herd immunity in an area of endemic infection. Over many rounds of treatment, this would increase reinfection rates (as individuals would not have the same level of past experience of infection and would therefore not gain the same level of immunity), which would act to lessen the long-term impact of preventive chemotherapy [6, 54]. This relationship could be further complicated if treatment induces acquired immunity [65]. There is also uncertainty regarding the biology of the long-term mating behaviour of the adult worms [66, 67] and therefore the most appropriate mating function to use within the models [6, 68]. Furthermore, it important to note that the model does not account for migration or animal reservoirs. In addition, the model’s structure implicitly assumes that hosts contribute infectious material to a single reservoir (that is shared for the entire population) and the degree of parasite aggregation (defined by the negative binomial probability distribution) is assumed to be fixed.

It should also be noted that the scenarios for the relative pre-control burden in adults were informed by the limited age-stratified infection intensity data available and that there may be settings that fall outside this range - including variation of the age at which infection intensity peaks. This highlights the need for more high quality fully cross-sectional data regarding schistosomiasis infection levels in all age classes but especially adults, particularly as the global goals shift to transmission elimination. These data are often lacking due to the programmatic and logistical difficulties of performing the current diagnostic tests in communities.

Currently, the model’s prevalence estimates do not account for which infections would be detectable with the available diagnostic tests. When adjusting for this, it is important to account for the fact that the sensitivity of the diagnostic tests, will likely decrease as infection intensity decreases, i.e. the sensitivity of the test is not a constant.

### Programmatic issues and considerations for future economic evaluations

Currently whether or not adults are targeted within schistosomiasis control programmes is based on the prevalence of infection in SAC [2]. However, in a study in Nigeria [69], the prevalence of infection in SAC, the age group where most monitoring and evaluation activities and data collection is focused, was not a successful indicator of the burden of infection in adults. The pre-control burden in adults will likely be driven by a number of local behavioural and cultural factors, and will, therefore, vary across different countries (and even different regions within countries). This makes it difficult to make a universal SAC infection prevalence threshold for switching to community-wide mass treatment. This further highlights the need for more cost-effective rapid diagnostic tests that allow adults to be sampled more feasibly in a programmatic context [70].

In January of 2012 (as part of the London Declaration on NTDs), Merck KGaA pledged to increase its praziquantel donation from 50 million to 250 million tablets a year for as long as needed [5, 71]. This donation has greatly increased the availability of praziquantel, but it is still less than one-half of the more than 500 million tablets needed annually to treat everyone (children and adults) at risk under the current thresholds for treatment [72]. Potential praziquantel shortages need to be considered when considering the cost of expanding the use of community-wide treatment.

It should be noted that some school-based treatment programmes are financed by the Ministries of Education of endemic countries (and not the Ministries of Health). This needs to be considered when interpreting the conclusions of any cost and cost-effectiveness analysis of switching to community-wide mass treatment - as the same funds may not always be available (which will significantly change the incremental cost of changing strategy).

A further important programmatic consideration for treating continuously across entire communities is the potential risk of drug resistance developing. The current reservoir of untreated worms in adults may be diluting any resistant gene pool in children and therefore expanding treatment could increase the risk of drug resistance. This issue needs careful monitoring with more research to define markers to track *via* molecular epidemiological studies [73].

One of the most urgent research needs for both schistosomiasis and the soil-transmitted helminths is for detailed costing studies that investigate how the delivery costs of preventive chemotherapy may change when switching to a community from a school-based treatment programme (as well as the potential costs of integrating treatment of adults into other control programmes) [74–76]. In addition, many NTD control programmes are now integrated, and in some settings they may have already established community-delivery platforms which could also be used for schistosomiasis treatment, allowing the treatment of adults at a smaller incremental cost. The potential integration of NTD programmes can have important implications regarding the cost-effectiveness of different strategies [8, 9].

Switching to a community-wide mass treatment strategy could be cost-saving in many settings in the long term - due to its capacity to break transmission with fewer treatment rounds (Fig. 7). However, when investigating these potential cost-savings it will be important to consider the risk of human migration resourcing infection; transmission models can be useful in evaluating the risk of this and potential management strategies. It will also be important to consider the implications of potential hybridization of different *Schistosoma* species, which could increase their geographical range if it changes which snail species are viable intermediate hosts making elimination more challenging [77–79], as well as the potential role of animal reservoirs [79, 80].

Further studies are needed to assess the coverage and compliance of different age groups achieved when using different treatment delivery methods [27]. It will also be important to investigate the costs (and achieved coverage) of targeting high-risk adults and how this compares to the costs of targeting the whole community. If a sufficient coverage of high-risk adults could be achieved, it could be a more cost-effective alternative to switching to community-wide mass treatment.

Within this paper, we did not evaluate the benefit of the treating Pre-SAC and this should be explored in future work.

It is also important to recognise that urogenital schistosomiasis may have a role in human immunodeficiency virus (HIV) and human papillomavirus (HPV) infections [81–83] and could potentially make co-infected individuals more infectious [82, 84]. This suggests that community-wide treatment for schistosomiasis could have benefits on other diseases - which are not captured in this study. The potential impact of schistosomiasis on other diseases should be researched further and considered in policy decisions.

## Conclusions

Community-wide mass treatment was found to be more effective for controlling schistosome infection than using a school-based programme only targeting SAC. However, its relative benefit was highly variable across the different scenarios explored. For example, the incremental impact of community-wide mass treatment relative to school-based treatment was very dependent on the local epidemiological setting and the method used to approximate the impact of treatment on morbidity, i.e. was the effectiveness metric based on reductions in infection prevalence or reductions in infection intensity. This has important implications regarding the generalisability of cost-effectiveness analyses of schistosomiasis interventions. Due to our limited understanding of the causal link between schistosomiasis morbidity and infection, we believe that caution should be employed when interpreting modelling results regarding the amount of schistosomiasis morbidity averted by different treatment strategies. In particular, our results highlight that basing the effectiveness/disease metric solely on reduced infection prevalence may produce misleading conclusions and that this area needs further research. It is important to highlight that although switching to regular annual community-wide mass treatment may not always be advisable, this does not mean that high-risk adults should not be targeted when possible. For areas where the goal is to eliminate transmission, the projected benefit of community-wide mass treatment was more consistent. Ultimately, whether community-wide mass treatment is appropriate will depend on the epidemiological and programmatic setting, i.e. the relative pre-control burden in adults, school enrolment and transmission intensity, and whether the goal is morbidity control or eliminating transmission. This work highlights the importance of not over-generalising conclusions and policy in this area, but of basing decisions on high-quality epidemiological data and quantitative analyses of the impact of interventions in a range of settings.

## Additional file

Additional file 1: Table S1. Model parameters for *Schistosoma mansoni.* **Table S2.** Model for the three age-intensity profiles. **Table S3.** Sensitivity of the relative increase in effectiveness when using annual community-wide versus school-based treatment to the treatment coverage in adults. **Table S4.** Sensitivity of the relative increase in effectiveness when using annual community-wide versus school-based treatment to the assumed mean life expectancy of the adult worms. **Table S5.** Sensitivity of the relative increase in effectiveness when using annual community-wide versus school-based treatment to the assumed level of systematic non-compliance. **Table S6.** Relative increase in effectiveness when using annual community-wide versus school-based treatment when assuming poor school enrolment. **Table S7.** The effect of using different fitting methods to account for the age-intensity profile on the projected incremental increase in effectiveness when using annual community-wide versus school-based treatment. **Figure S1.** The impact of annual school-based treatment on the mean worm burden in different age groups in three settings with a different relative pre-control worm burden in adults. **Figure S2.** Sensitivity of the number of years of annual treatment to achieve elimination of *Schistosoma mansoni* to the assumed level of systematic non-compliance. **Figure S3.** Sensitivity of the number of years of annual treatment to achieve elimination of *Schistosoma mansoni* to the assumed mean life expectancy of the worms. (PDF 1366 kb)

## Abbreviations

DALY: Disability-adjusted life year
Epg: Eggs per gram of stool
GBD: Global burden of disease
HIV: Human immunodeficiency virus
HPV: Human papillomavirus
MDA: Mass drug administration
NTD: Neglected tropical disease
R_0_: Basic reproduction number
SAC: School-aged children
WHO: World Health Organization
