# Supplementary material for: Evaluating the variation in the projected benefit of community-wide mass treatment for schistosomiasis: Implications for future economic evaluations
Source: Parasit Vectors. 2017 Apr 28;10:213. doi: 10.1186/s13071-017-2141-5 (PMC5410074; doi:10.1186/s13071-017-2141-5)
Supplement: Additional file 1: Table S1. — Model parameters for Schistosoma mansoni. Table S2. Model for the three age-intensity profiles. Table S3. Sensitivity of the relative increase in effectiveness when using annual community-wide versus school-based treatment to the treatment coverage in adults. Table S4. Sensitivity of the relative increase in effectiveness when using annual community-wide versus school-based treatment to the assumed mean life expectancy of the adult worms. Table S5. Sensitivity of the relative increase in effectiveness when using annual community-wide versus school-based treatment to the assumed level of systematic non-compliance. Table S6. Relative increase in effectiveness when using annual community-wide versus school-based treatment when assuming poor school enrolment. Table S7. The effect of using different fitting methods to account for the age-intensity profile on the projected incremental increase in effectiveness when using annual community-wide versus school-based treatment. Figure S1. The impact of annual school-based treatment on the mean worm burden in different age groups in three settings with a different relative pre-control worm burden in adults. Figure S2. Sensitivity of the number of years of annual treatment to achieve elimination of Schistosoma mansoni to the assumed level of systematic non-compliance. Figure S3. Sensitivity of the number of years of annual treatment to achieve elimination of Schistosoma mansoni to the assumed mean life expectancy of the worms. (PDF 1366 kb) [file 13071_2017_2141_MOESM1_ESM.pdf]

Supplementary information

The Model

The fundamental model used to describe the mean worm burden of individuals of a given age and the quantity of infectious eggs in the environment was developed from the founding work of Anderson and May [1]. The current version of the model is described in detail in [2-5]. The key parameters in Supporting Table 1 and Supporting Table 2.

The model was further adapted to allow for systematic non-compliance (individuals never taking treatment) [6, 7]. A more detailed description of the model is provided in Truscott *et al*. [8].

Supporting Table S1: Model parameters for *Schistosoma mansoni*

Parameter	Value	Source
**Adult worm life expectancy**	5.71 years	[9, 10]
**Aggregation parameter, *k***	0.24	[11]
**Density dependence fecundity, γ**	0.0007/female worm	[3]
**Drug efficacy (proportion of worms killed by praziquantel)**	0.86	[12]
**Egg output per female worm in absence of density dependence (in terms of faecal egg counts)**	0.14	[13]
**Human demography**	Based on Uganda's demographical profile	[14, 15]
**Life expectancy of the infected snails**	4 weeks	[16]

Baseline scenarios

Based on the available data [3], we developed three age-intensity profiles representing a range of possible scenarios regarding the relative burden in adults.

When changing the assumed the life span of the adult worms, the age-specific parameters for the exposure and contribution to the infectious reservoir were refitted – so that the shape of the age profile does not change.

Supporting Table S2: Model for the three age-intensity profiles

Parameter	Setting 1: Low burden in adults	Setting 2: Medium burden in adults	Setting 3: High burden in adults
<u>5.71 year average lifespan</u>			
**Basic Reproductive number (Lower setting)**	1.30485	1.31547	1.30577
**Basic Reproductive number (Higher setting)**	1.6687	1.68	1.62886
**Age specific degree of exposure and contribution to the infectious reservoir**	0-5 year olds: 0.01<br>5-10 year olds: 1.2<br>10-16 year olds: 1<br>16+ year olds: 0.02	0-5 year olds: 0.032<br>5-10 year olds: 0.61<br>10-16 year olds: 1<br>16+ year olds: 0.06	0-5 year olds: 0.01<br>5-12 year olds: 0.61<br>12-20 year olds: 1<br>20+ year olds: 0.12
<u>4 year average lifespan</u>			
**Basic Reproductive number (Lower setting)**	1.33155	1.3638	1.325568
**Basic Reproductive number (Higher setting)**	1.7752	1.83495	1.6778
**Age specific degree of exposure and contribution to the infectious reservoir**	0-5 year olds: 0.32<br>5-10 year olds: 4.95<br>10-16 year olds:1<br>16+ year olds: 0.065	0-5 year olds: 0.032<br>5-10 year olds: 0.14<br>10-16 year olds: 1<br>16+ year olds: 0.09	0-5 year olds: 0.01<br>5-12 year olds: 0.5<br>12-20 year olds: 1<br>20+ year olds: 0.14

The model was used to simulate two transmission settings; a higher transmission setting with an age-weighted mean worm burden of 155, and a lower transmission setting with a mean worm burden of 60. The data used to define the three scenarios is presented in [3].

The model was used to simulate two transmission settings; a higher transmission setting with an age-weighted mean worm burden of 155 (based on model fits to the data [5]), and a lower transmission setting with a mean worm burden of 60. To ensure the results for the different scenarios are comparable, the R_0 was adjusted such that the different scenarios had the same pre-control mean worm burden (i.e. we ensured that we are not comparing the impact of both a different age-infection profile and a different pre-control burden when comparing the different scenarios).

Supporting Table S3: Sensitivity of the relative increase in effectiveness when using annual community-wide versus school-based treatment to the treatment coverage in adults (55% vs. 75%).

Metric	Relative pre-control worm burden in adults	Relative increase in effectiveness	
		Higher transmission setting	Lower transmission setting
Worm-years averted	Low	15-17%	13-15%
	Medium	27-30%	20-23%
	High	72-80%	55-59%
Prevalent case years averted	Low	68-83%	59-70%
	Medium	101-124%	83-99%
	High	228-303%	204-258%
Heavy case years averted	Low	21-23%	11-12%
	Medium	39-43%	16-18%
	High	107-118%	49-52%

The range in each cell shows the variation in the relative increase in effectiveness when using annual community-wide versus school-based treatment to the assumed level of treatment coverage in adults (55% vs. 75%). The scenarios for the relative pre-control burden in adults are shown in Figure 2 (note they have the same age-weighted overall mean worm burden). The results assume 5% systematic non-compliance and 75% treatment coverage of SAC. The analysis was conducted with a five-year implementation period and a 15-year time horizon (i.e. looking at the impact of five years of treatment over 15 years).

Supporting Table S4: Sensitivity of the relative increase in effectiveness when using annual community-wide versus school-based treatment to the assumed mean life expectancy of the adult worms (4 years vs. 5.71 years).

Metric	Relative pre-control worm burden in adults	Relative increase in effectiveness	
		Higher transmission setting	Lower transmission setting
Worm-years averted	Low	7-17%	5-15%
	Medium	30-55%	23-30%
	High	80-94%	59-61%
Prevalent case years averted	Low	70-83%	47-70%
	Medium	124-182%	99-149%
	High	303-359%	258-302%
Heavy case years averted	Low	10-23%	4-12%
	Medium	43-83%	18-26%
	High	118-139%	52-54%

The range in each cell shows the variation in the relative increase in effectiveness when using annual community-wide versus school-based treatment to the assumed mean life expectancy of the adult worms (4 years vs. 5.71 years). The scenarios for the relative pre-control burden in adults are shown in Figure 2 (note they have the same age-weighted overall mean worm burden). The results assume a treatment coverage of 75% and 5% systematic non-compliance. The analysis was conducted with a five-year implementation period and a 15-year time horizon (i.e. looking at the impact of five years of treatment over 15 years).

Supporting Table S5: Sensitivity of the relative increase in effectiveness when using annual community-wide versus school-based treatment to the assumed level of systematic non-compliance (0% vs 20%).

Metric	Relative pre-control worm burden in adults	Relative increase in effectiveness	
		Higher transmission setting	Lower transmission setting
Worm-years averted	Low	16-17%	14-15%
	Medium	28-30%	22-22%
	High	76-81%	59-60%
Prevalent case years averted	Low	58-124%	49-98%
	Medium	74-195%	60-252%
	High	178-491%	154-408%
Heavy case years averted	Low	21-25%	12-13%
	Medium	40-42%	17-21%
	High	111-114%	48-60%

The range in each cell shows the variation in the relative increase in effectiveness when using annual community-wide versus school-based treatment to the assumed level of systematic non-compliance (0% vs. 20%). The scenarios for the relative pre-control burden in adults are shown in Figure 2 (note they have the same age-weighted overall mean worm burden). The results assume a treatment coverage of 75%. The analysis was conducted with a five-year implementation period and a 15-year time horizon (i.e. looking at the impact of five years of treatment over 15 years).

Supporting Table S6: Relative increase in effectiveness when using annual community-wide versus school-based treatment when assuming poor school enrolment.

Metric	Relative pre-control worm burden in adults	Relative increase in effectiveness	
		Higher transmission setting	Lower transmission setting
Worm-years averted	Low	32%	23%
	Medium	45%	33%
	High	98%	74%
Prevalent case years averted	Low	169%	146%
	Medium	205%	168%
	High	381%	330%
Heavy case years averted	Low	51%	18%
	Medium	70%	26%
	High	149%	67%

The results assume the systematic non-compliance rate is 20% for the school-based programme, and 5% when using community-wide mass treatment (simulating a scenario where many of the non-enrolled SAC are only reached when using a community-based programme). The scenarios for the relative pre-control burden in adults are shown in Figure 2 (note they have the same age-weighted overall mean worm burden). The treatment coverage was assumed to be 75%. The analysis was conducted with a five-year implementation period and a 15-year time horizon (i.e. looking at the impact of five years of treatment over 15 years).

Supporting Table S7: The effect of using different fitting methods to account for the age-intensity profile on the projected incremental increase in effectiveness when using annual community-wide versus school-based treatment.

	**When fitting to fully age structured data**	**When only fitting to the mean burdens in children and adults**	**Percentage difference between the two fitting methods**
Average number of worm-years averted per person	645.38	830.58	29%
Prevalent case years averted (per 100 individuals)	308.39	439.36	42%
Heavy case years averted (per 100 individuals)	56.43	77.45	31%

In the first results column the model was fitted to fully age-structured data (and therefore accounts for the true shape of the age-intensity profile) *[5]. In the second results column, the model was only fitted to reproduce the estimated mean pre-control worm burdens in SAC and adults from the same dataset. The data used in this example is from the Iietune village (Kenya) [60]. The results assume a treatment coverage of 75% and no systematic non-compliance. The analysis was conducted with a five-year implementation period and a 15-year time horizon (i.e. looking at the effect of five years of treatment for 15 years).*

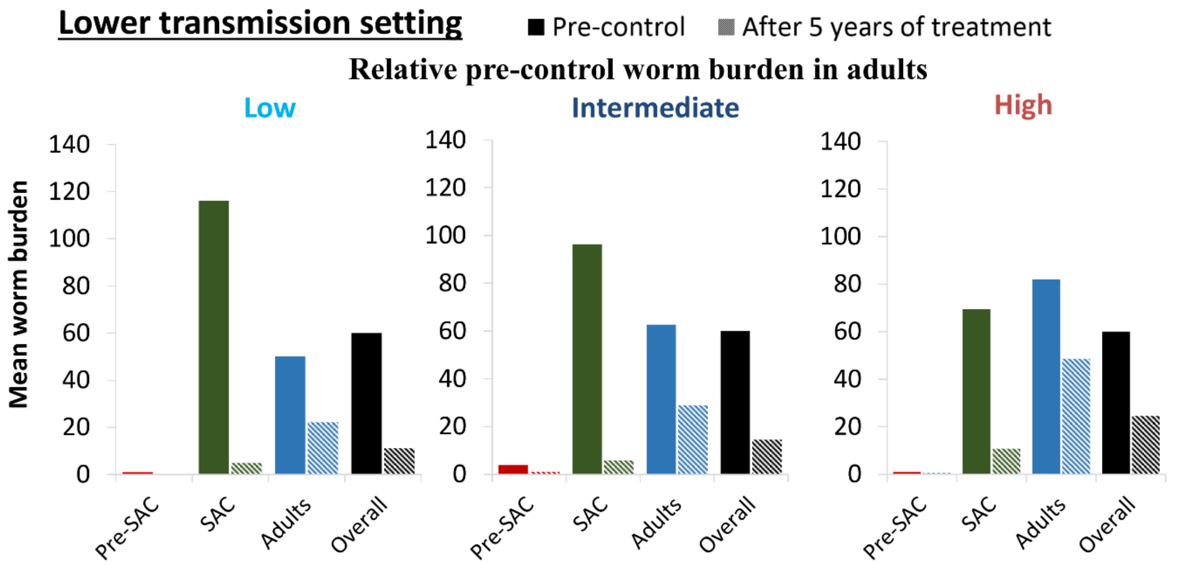

Supporting Figure S1: The impact of annual school-based treatment on the mean worm burden in different age groups in three settings with a different relative pre-control worm burden in adults. *The solid bars represent the pre-control burden and the hashed bars, the burden after 5 years of treatment. The scenarios for the relative pre-control burden in adults are shown in Figure 2 (note they have the same age-weighted overall mean worm burden). The results assume a treatment coverage of 75% and 5% systematic non-compliance. The results pertaining to the higher transmission setting are shown in Figure 4. Pre-SAC: 2-4 year-olds, SAC: 5-14 year-olds and adults: ≥15 year-olds.*

Relative pre-control worm burden in adults

20% systematic non-compliance

Low

Coverage of SAC (%) \ Coverage of adults (%)	0%	20%	40%	60%	80%	95%
0%	NA	NA	NA	NA	NA	-
20%	NA	NA	NA	NA	NA	-
40%	10	10	10	9	9	-
60%	7	7	7	7	7	-
80%	6	6	6	6	6	-
95%	-	-	-	-	-	-

Intermediate

Coverage of SAC (%) \ Coverage of adults (%)	0%	20%	40%	60%	80%	95%
0%	NA	NA	NA	NA	NA	-
20%	NA	NA	NA	NA	NA	-
40%	12	10	10	10	9	-
60%	9	8	7	7	7	-
80%	8	7	6	6	6	-
95%	-	-	-	-	-	-

High

Coverage of SAC (%) \ Coverage of adults (%)	0%	20%	40%	60%	80%	95%
0%	NA	NA	NA	NA	NA	-
20%	NA	NA	13	11	10	-
40%	NA	11	9	8	7	-
60%	14	9	7	6	6	-
80%	12	8	6	6	5	-
95%	-	-	-	-	-	-

0% systematic non-compliance

Low

Coverage of SAC (%) \ Coverage of adults (%)	0%	20%	40%	60%	80%	95%
0%	NA	NA	NA	NA	NA	NA
20%	NA	NA	NA	15	15	15
40%	8	7	7	7	7	7
60%	5	5	4	4	4	4
80%	3	3	3	3	3	3
95%	3	2	2	2	2	2

Intermediate

Coverage of SAC (%) \ Coverage of adults (%)	0%	20%	40%	60%	80%	95%
0%	NA	NA	NA	NA	NA	NA
20%	NA	NA	15	14	14	13
40%	10	8	7	7	7	7
60%	6	5	5	4	4	4
80%	5	4	3	3	3	3
95%	4	3	3	2	2	2

High

Coverage of SAC (%) \ Coverage of adults (%)	0%	20%	40%	60%	80%	95%
0%	NA	NA	NA	NA	15	14
20%	NA	14	10	8	7	7
40%	15	9	6	5	5	4
60%	11	6	5	4	3	3
80%	9	5	4	3	3	2
95%	8	5	3	3	2	2

Supporting Figure S2: Sensitivity of the number of years of annual treatment to achieve elimination of *Schistosoma mansoni* to the assumed level of systematic non-compliance. *The scenarios for the relative pre-control burden in adults are shown in Figure 2 (note they have the same age-weighted overall mean worm burden). The results use the parameters for the lower transmission setting. When assuming a systematic non-compliance rate of 20% it is not possible to get 95% coverage (indicated by a dash on the figure). NA; Not achievable within 15 years of annual treatment.*

Relative pre-control worm burden in adults

Assuming a mean worm life expectancy of 4 years

Low

Coverage of SAC (%)	Coverage of adults (%) 0%	20%	40%	60%	80%	95%
0%	NA	NA	NA	NA	NA	NA
20%	NA	NA	NA	NA	NA	NA
40%	11	10	10	10	10	10
60%	7	7	6	6	6	6
80%	5	5	5	5	5	5
95%	4	4	4	4	4	4

Intermediate

Coverage of SAC (%)	Coverage of adults (%) 0%	20%	40%	60%	80%	95%
0%	NA	NA	NA	NA	NA	NA
20%	NA	NA	NA	NA	NA	NA
40%	NA	12	10	9	9	8
60%	11	8	7	6	6	6
80%	9	6	5	5	4	4
95%	8	6	5	4	3	3

High

Coverage of SAC (%)	Coverage of adults (%) 0%	20%	40%	60%	80%	95%
0%	NA	NA	NA	NA	NA	NA
20%	NA	NA	11	9	8	7
40%	NA	10	7	6	5	5
60%	13	8	6	5	4	4
80%	11	7	5	4	3	3
95%	10	6	4	3	3	2

Assuming a mean worm life expectancy of 5.71 years (baseline assumption)

Low

Coverage of SAC (%)	Coverage of adults (%) 0%	20%	40%	60%	80%	95%
0%	NA	NA	NA	NA	NA	NA
20%	NA	NA	NA	NA	15	15
40%	8	8	8	8	7	7
60%	5	5	5	5	5	5
80%	4	4	3	3	3	3
95%	3	3	3	3	2	2

Intermediate

Coverage of SAC (%)	Coverage of adults (%) 0%	20%	40%	60%	80%	95%
0%	NA	NA	NA	NA	NA	NA
20%	NA	NA	NA	15	14	14
40%	10	9	8	7	7	7
60%	7	6	5	5	5	5
80%	5	4	4	3	3	3
95%	5	4	3	3	3	2

High

Coverage of SAC (%)	Coverage of adults (%) 0%	20%	40%	60%	80%	95%
0%	NA	NA	NA	NA	NA	15
20%	NA	15	11	9	8	7
40%	NA	9	7	6	5	5
60%	12	7	5	4	4	3
80%	10	6	4	3	3	3
95%	9	5	4	3	2	2

Supporting Figure S3: Sensitivity of the number of years of annual treatment to achieve elimination of *Schistosoma mansoni* to the assumed mean life expectancy of the worms. *The scenarios for the relative pre-control burden in adults are shown in Figure 2 (note they have the same age-weighted overall mean worm burden). The results use the parameters for the lower transmission setting, and assume 5% systematic non-compliance. NA; Not achievable within 15 years of annual treatment.*

Reference

1. Anderson RM, May RM: Infectious diseases of humans : dynamics and control. Oxford ; New York: Oxford University Press; 1991.
2. Truscott JE, Hollingsworth TD, Brooker SJ, Anderson RM: Can chemotherapy alone eliminate the transmission of soil transmitted helminths? *Parasit Vectors* 2014, 7(1):266.
3. Anderson RM, Turner, H.C., Farrell, S.H. and Truscott, J.E.: Studies of the transmission dynamics, mathematical model development, and the control of schistosome parasites by mass drug administration in human communities. *Adv Parasitol* 2016, 94:199-246.
4. Truscott JE, Turner HC, Farrell SH, Anderson RM: Soil Transmitted Helminths: mathematical models of transmission, the impact of mass drug administration and transmission elimination criteria. *Adv Parasitol* 2016, 94:133-198.
5. Anderson R, Turner H, Farrell S, Yang J, Truscott J: What is required in terms of mass drug administration to interrupt the transmission of schistosome parasites in regions of endemic infection? *Parasit Vectors* 2015, 8(1):1-11.
6. Truscott JE, Turner HC, Anderson RM: What impact will the achievement of the current World Health Organisation targets for anthelmintic treatment coverage in children have on the intensity of soil transmitted helminth infections? *Parasit Vectors* 2015, 8(1):551.
7. Farrell S, Truscott JE, Anderson RM: The importance of patient compliance in repeated rounds of mass drug administration (MDA) for the elimination of intestinal helminth transmission. *Parasit Vectors* 2017, In press.
8. Truscott, J.E., Gurarie. D., Alsallaq, R., Toor, J., Yoon, N., Farrell, S.H., Turner, H.C., Phillips, A.E., Aurelio, H.O., Ferro, J., King, C.H., and Anderson, R.M (2017). A comparison of two mathematical models of the impact of mass drug administration on the transmission and control of schistosomiasis. Epidemics, 18, 29-37.
9. Anderson RM, Medley GF: Community control of helminth infections of man by mass and selective chemotherapy. *Parasitology* 1985, 90:629-660.
10. Fulford AJ, Butterworth AE, Ouma JH, Sturrock RF: A statistical approach to schistosome population dynamics and estimation of the life-span of Schistosoma mansoni in man. *Parasitology* 1995, 110 (Pt 3):307-316.
11. Chan MS, Guyatt HL, Bundy DA, Booth M, Fulford AJ, Medley GF: The development of an age structured model for schistosomiasis transmission dynamics and control and its validation for Schistosoma mansoni. *Epidemiol Infect* 1995, 115(2):325-344.
12. Zwang J, Olliaro PL: Clinical efficacy and tolerability of praziquantel for intestinal and urinary schistosomiasis-a meta-analysis of comparative and non-comparative clinical trials. *PLoS neglected tropical diseases* 2014, 8(11):e3286.
13. de Vlas SJ, Nagelkerke NJ, Habbema JD, van Oortmarssen GJ: Statistical models for estimating prevalence and incidence of parasitic diseases. *Stat Methods Med Res* 1993, 2(1):3-21.
14. Pullan RL, Kabatereine NB, Quinnell RJ, Brooker S: Spatial and genetic epidemiology of hookworm in a rural community in Uganda. *PLoS Negl Trop Dis* 2010, 4(6):e713.
15. Anderson RM, Truscott JE, Hollingsworth TD: The coverage and frequency of mass drug administration required to eliminate persistent transmission of soil-transmitted helminths. *Philos Trans R Soc Lond B Biol Sci* 2014, 369(1645):20130435.
16. Anderson RM, May RM: Prevalence of schistosome infections within molluscan populations: observed patterns and theoretical predictions. *Parasitology* 1979, 79(01):63-94.
